# Mycoplasmal conjunctivitis in wild songbirds: the spread of a new contagious disease in a mobile host population.

**DOI:** 10.3201/eid0301.970110

**Published:** 1997

**Authors:** J R Fischer, D E Stallknecht, P Luttrell, A A Dhondt, K A Converse

**Affiliations:** University of Georgia, Athens, USA.

## Abstract

A new mycoplasmal conjunctivitis was first reported in wild house finches (Carpodacus mexicanus) in early 1994. The causative agent was identified as Mycoplasma gallisepticum (MG), a nonzoonotic pathogen of poultry that had not been associated with disease in wild songbirds. Since the initial observations of affected house finches in the mid-Atlantic region, the disease has become widespread and has been reported throughout the eastern United States and Canada. By late 1995, mycoplasmal conjunctivitis had spread to an additional species, the American goldfinch (Carduelis tristis). This new disease exemplifies the rapid spread of a pathogen following introduction into a mobile wildlife population and provides lessons that may apply to emerging human diseases.


### Mycoplasmal Conjunctivitis in Wild Songbirds: The Spread of a New Contagious Disease in a Mobile Host Population

A new mycoplasmal conjunctivitis was first reported in wild house finches (*Carpodacus mexicanus*) in early 1994. The causative agent was identified as *Mycoplasma gallisepticum* (MG), a nonzoonotic pathogen of poultry that had not been associated with disease in wild songbirds. Since the initial observations of affected house finches in the mid-Atlantic region, the disease has become widespread and has been reported throughout the eastern United States and Canada. By late 1995, mycoplasmal conjunctivitis had spread to an additional species, the American goldfinch (*Carduelis tristis*). This new disease exemplifies the rapid spread of a pathogen following introduction into a mobile wildlife population and provides lessons that may apply to emerging human diseases.

In February 1994, house finches with swollen or crusty eyelids and impaired vision were observed at backyard bird feeders in suburban Washington, D.C. (1). Severely affected birds lingered on bird feeders or on the ground surrounding the feeders. These birds had chronic lymphoplasmacytic conjunctivitis, sinusitis, and rhinitis. Microorganisms resembling mycoplasmas were adhering to conjunctival epithelium, and *Mycoplasma gallisepticum* (MG) was isolated from affected tissues (2,3). A Maryland field survey of MG in house finches in late 1994 showed a strong association between conjunctivitis and MG by culture and polymerase chain reaction (PCR) (3). Subsequently the disease was reproduced by inoculation of unaffected house finches with a finch-derived MG isolate (Fischer, unpublished data).

Since the first reports from the mid-Atlantic states, mycoplasmal conjunctivitis in house finches has spread rapidly to the north, south, and west. The disease was first monitored by wildlife biologists with state and federal wildlife resource agencies, and by October 1994, affected house finches had been reported in nine states in the mid-Atlantic region (Figure). Beginning in November 1994, backyard sightings of healthy and diseased house finches have been recorded by private citizens participating in a survey conducted by the Cornell Laboratory of Ornithology (4). The percentage of participants reporting diseased house finches has steadily increased since the survey began: House finches with conjunctivitis were reported by 11% of 1,413 participants in November 1994, by 17.3% of 1,239 participants in March 1995, by 28.1% of 769 participants in November 1995, and by 35.8% of 1,047 participants in March 1996. Most reports in November 1994 came from the mid-Atlantic region, and 30% to 40% of survey participants in this area had reported diseased finches through March 1996. The survey also has documented the dramatic spread of disease to house finches in the Midwest and Southeast (Figure). In November 1994, diseased finches were reported by only 0.4% of 229 participants in these regions, but by March 1996, the percentage had climbed to 37% of 257 participants. Mycoplasmal conjunctivitis now has been reported almost throughout the eastern population of house

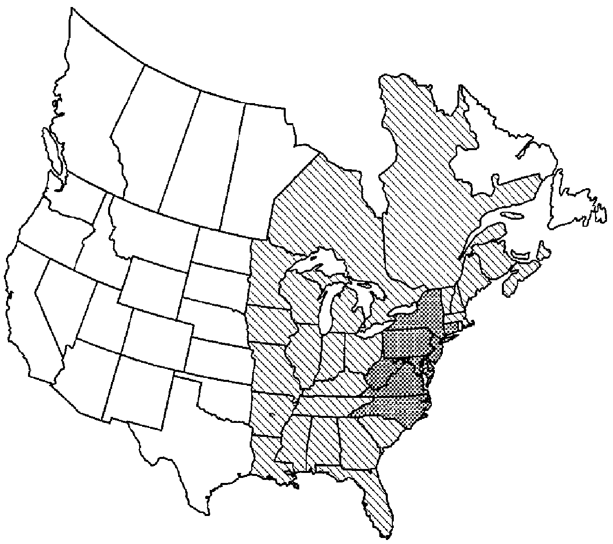

▩ States reporting diseased house finches through October 1994

▨ Additional states and provinces reporting diseased house finches through June 1996

Figure. Case distribution of house finches with conjunctivitis, October 1994–June 1996.

Table. Test results for *Mycoplasma gallisepticum* in house finches and American goldfinches, Southeastern Cooperative Wildlife Disease Study, May 1994–June 1996

| | | | Conjunctivitis | | | *Mycoplasma gallisepticum*[a] | | |
|---|---|---|---|---|---|---|---|---|
| State of Origin | Species | Number | Bilateral | Unilateral | Rhinitis | Isolation | PCR | Serology[b] |
| AL | House finch | 1 | 1 | 0 | 1 | 0/1 | 1/1 | 0/0 |
| GA | House finch | 6 | 3 | 3 | 2 | 3/6 | 3/3 | 6/6 |
| | Am. goldfinch | 2 | 1 | 1 | 0 | 0/2 | 2/2 | 2/2 |
| KY | House finch | 2 | 1 | 1 | 2 | 1/2 | 2/2 | 2/2 |
| MD | House finch | 9 | 4 | 5 | 6 | 0/7 | 6/6 | 6/6 |
| | Am. goldfinch | 1 | 1 | 0 | 0 | 0/1 | 1/1 | 1/1 |
| OH | House finch | 1 | 1 | 0 | 1 | 1/1 | 0/0 | 0/0 |
| SC | Am. goldfinch | 1 | 1 | 0 | 0 | 0/1 | 1/1 | 0/0 |
| TN | House finch | 12 | 9 | 3 | 6 | 2/7 | 2/2 | 4/6 |
| | Am. goldfinch | 1 | 1 | 0 | 1 | 0/1 | 0/1 | 0/0 |
| TOTALS | House finch | 31 | 19 | 12 | 18 | 7/24 | 14/14 | 18/20 |
| | Am. Goldfinch | 5 | 3 | 1 | 1 | 0/5 | 4/5 | 3/3 |

[a] Data are presented as number positive/number tested.
[b] Positive = 2+ on rapid plate agglutination test.

finches. No diseased birds have been reported within the western population of house finches, which occupies the historic range of this species.

House finches are native to the western United States, where they were captured during the 1930s for sale in eastern pet shops. House finches were released in New York City in the early 1940s when regulations regarding the commercial trade of domestic songbirds changed. The first nesting pair of house finches was observed on Long Island in 1943, and by 1951 the area held an estimated 280 birds (5). The eastern house finch population has expanded dramatically from the original limited number of birds in New York; it currently contains millions of finches over the entire eastern and midwestern United States and southeastern Canada (6).

Mycoplasmal conjunctivitis apparently has spread to another wild finch species in the eastern United States. During the winter and spring of 1995 to 1996, American goldfinches with inflamed eyelids were reported within the range of diseased house finches in Georgia, Maryland, North Carolina, South Carolina, and Tennessee. The goldfinches had lesions identical to those of house finches with mycoplasmal conjunctivitis, and in March 1996, MG was isolated from two diseased goldfinches from North Carolina (7).

House finches and goldfinches were submitted for diagnostic laboratory examinations during this epornitic. At the Southeastern Cooperative Wildlife Disease Study, gross and microscopic postmortem examinations, serologic tests for antibodies against MG (3), mycoplasmal cultures, and PCR testing for MG (3) were performed on 31 house finches and five goldfinches from seven states (Table). Consistent pathologic findings in both species included moderate to severe lymphoplasmacytic conjunctivitis often with hyperplasia of associated lymphoid and epithelial tissues, and rhinitis. Occasionally, keratitis and tracheitis were observed. Antibodies against MG were detected by rapid plate agglutination tests in 91% of tested birds, and positive PCR products were obtained from 95% of birds tested. MG was isolated from only 24% of birds cultured; this percentage probably reflects the fastidiousness of the strain affecting wild finches and the condition of the specimens.

MG is a major pathogen of domestic poultry and causes infectious sinusitis in turkeys, chronic respiratory disease in chickens, and subclinical infections (8). MG also has been associated with conjunctivitis in chickens (9) and farmed gamebirds (10). Clinical disease had not been associated with MG in wild passerine birds, although MG (11) and antibodies against MG (12) have been detected in these birds. Infections in poultry persist despite antimicrobial treatment or the development of antibody response; MG can be transmitted by direct contact, by airborne droplets or dust, and vertically through eggs (8). Like other mycoplasmas, MG can alter its antigenic surface components in vivo. This feature may contribute to its ability to persistently infect by adapting to the host environment and evading the host immune response (13). The possible role of this antigenic variation in the adaptation to new host species is unknown.

The source of the MG affecting house finches and goldfinches and its introduction into the eastern house finch population are under investigation. Field isolates of MG collected during 1994 through early 1996 from house finches, goldfinches, and domestic poultry; three poultry vaccine strains of MG; and three poultry reference strains of MG were analyzed by random amplification of polymorphic DNA (7). Molecular characterization showed that isolates from both finch species over a wide geographic range had identical or nearly identical random amplification of polymorphic DNA banding patterns, but they differed from reference strains, vaccine strains, and isolates from commercial poultry (7). These results suggested that the finch epornitic arose from a single source and was caused by an MG strain that differed from strains commonly associated with poultry disease or vaccination. The ultimate source of the outbreak strain remains unknown; however, molecular studies have not shown any relationship between wild finch and poultry strains of MG (7). Possible sources of MG include unrecognized reservoirs of MG in the wild or small poultry operations with poor biosecurity, such as backyard chicken flocks.

The remarkable spread of mycoplasmal conjunctivitis in wild finches probably reflects both bird behavior and human activity. House finches are well adapted to human land use practices; they nest and feed in open areas around buildings and farms, as well as in suburban settings with ornamental trees and shrubbery. They eat weed and grass seeds, as well as fruits and berries of flowering trees and shrubs. During the winter, house finches flock around backyard bird feeders (5). Although precise MG transmission modes are unknown, the propensity of this highly gregarious species to assemble at bird feeders may enhance contact with infected birds or with surfaces contaminated with the causative agent. Unlike their western counterparts, house finches within the eastern range of the species may migrate several hundred miles (14) and thus disseminate an infectious agent over a large geographic area.

Intentional and unintentional ecologic changes may have contributed significantly to the current mycoplasmal conjunctivitis outbreak. The affected species was introduced into a new range, and the birds have flourished because they prefer the areas that humans increasingly provide. Although the eastern house finch population is large and widely distributed, the limited genetic pool from which it descended may have contributed to its apparently high susceptibility to MG. The causative agent is best known for its association with domestic poultry, and its spread through house finches appears to have been enhanced by bird feeders, which not only provide an opportunity for increased contact between infected and uninfected birds, but also may prolong the lives of infectious, diseased birds that otherwise would not have been able to feed. The combination of these and other unknown factors has resulted in the emergence of a severe infectious disease that has spread rapidly through the susceptible host population and could become permanently established in house finches and possibly other species.

Even though human activity has contributed to the emergence of this disease, human efforts to control it have not been successful. Treatment and immunization strategies to control infectious human and domestic animal diseases are not available, and if they were available, they would likely be impractical and costly. Treatment and subsequent release of individual birds in rehabilitation facilities is of questionable value because the effect may be minimal on a population basis. Furthermore, it is unknown if birds released after treatment remain persistently infected with MG. Moreover, the presence of multiple avian species in a rehabilitation clinic may enhance the transmission of MG to other species (2).

The current epornitic of mycoplasmal conjunctivitis in house finches has some interesting parallels to emerging human diseases, particularly regarding the substantial roles that human activity, ecologic changes, and the introduction of exotic species may play in the emergence of infectious disease. Additionally, the impressive speed at which this pathogen moved through the house finch population illustrates how rapidly a pathogen can be disseminated throughout a large geographic area within a highly gregarious and mobile host population. Finally, the lack of suitable control methods for MG in house finch populations and the inability to correct the ecologic conditions that contributed to its emergence provide strong support for preventive rather than reactive measures in dealing with the next potential wildlife or zoonotic disease.

### Acknowledgments

We thank the National Biological Service, U.S. Fish and Wildlife Service, Cornell Laboratory of Ornithology, as well as the state wildlife resource agencies, colleges of veterinary medicine, veterinary diagnostic laboratories, wildlife rehabilitation facilities, and private landowners who helped with diagnostic investigations and surveillance. This work was sponsored by the fish and wildlife agencies in Alabama, Arkansas, Florida, Georgia, Kentucky, Louisiana, Maryland, Mississippi, Missouri, North Carolina, Puerto Rico, South Carolina, Tennessee, Virginia, and West Virginia. Funds were provided by the Federal Aid to Wildlife Restoration Act (50 Stat. 917) and the Grant Agreement 14-45-0009-94-906, National Biological Service, U.S. Department of the Interior. Support also was received through Cooperative Agreement 95-9613-0032-CA, Veterinary Services, Animal and Plant Health Inspection Service, U.S. Department of Agriculture. Dr. Andre Dhondt was supported by the New York State Hatch Fund.

**John R. Fischer,* David E. Stallknecht,* M. Page Luttrell,* Andre A. Dhondt,† and Kathryn A. Converse‡**

*Southeastern Cooperative Wildlife Disease Study, The University of Georgia, Athens, Georgia, USA; †Cornell Laboratory of Ornithology, Ithaca, New York, USA; ‡National Wildlife Health Center, National Biological Service, Madison, Wisconsin, USA

### References

1. Fischer JR. Conjunctivitis in house finches. Proceedings of the 98th Annual Meeting of the USAHA; 1994 Oct 29-Nov 4; Grand Rapids (MI). Richmond (VA): U.S. Animal Health Association, 1994:530-1.
2. Ley DH, Berkhoff JE, McLaren JM. Isolation of *Mycoplasma gallisepticum* from house finches with conjunctivitis. Avian Dis 1996;40:480-3.
3. Luttrell MP, Fischer JR, Stallknecht DE, Kleven SH. Field investigations of *Mycoplasma gallisepticum* in house finches (*Carpodacus mexicanus*) from Maryland and Georgia. Avian Dis 1996;40:335-41.
4. Dhondt AA. Finch disease update. Birdscope 1996;10:4.
5. Elliot JJ, Arbib RS Jr. Origin and status of the house finch in the eastern United States. Auk 1953;70:31-7.
6. Hill GE. House finch (*Carpodacus mexicanus*). In: Poole A, Gill F, editors. Birds of North America, No. 46. Philadelphia: The Academy of Natural Sciences; Washington (DC): The American Ornithologists Union, 1993:1-23.
7. Ley DH, Berkhoff JE, Joyner K, Powers L, Levinsohn S. Molecular epidemiological investigations of *Mycoplasma gallisepticum* conjunctivitis in songbirds by random amplified polymorphic DNA (RAPD) analyses. Presented at the 11th International Congress of the International Organization for Mycoplasmology, Orlando, FL; 1996 July 14-19; Programs and Abstracts, pp. 53-4.
8. Yoder HS Jr. *Mycoplasma gallisepticum* infection. In: Calnek BW, Barnes HJ, Beard CW, Reid WM, Yoder HW Jr., editors. Diseases of poultry, 9th ed. Ames (IA): Iowa State Press, 1991:198-212.
9. Nunoya T, Yagihashi T, Tajima M, Nagasawa Y. Occurrence of keratoconjunctivitis apparently caused by *Mycoplasma gallisepticum* in layer chickens. Vet Pathol 1995;32:11-8.
10. Cookson, KC, Shivasprasad HL. *Mycoplasma gallisepticum* infection in chukar partridges, pheasants, and peafowl. Avian Dis 1994;38:914-21.
11. Shimizu T, Numano K, Uchida K. Isolation and identification of mycoplasmas from various birds: an ecological study. Japanese Journal of Veterinary Science 1979;41:273-82.
12. Stallknecht DE, Johnson DC, Emory WH, Kleven SH. Wildlife surveillance during a *Mycoplasma gallisepticum* epornitic in domestic turkeys. Avian Dis 1982;26:883-90.
13. Levisohn S, Rosengarten R, Yogev D. *In vivo* variation of *Mycoplasma gallisepticum* antigen expression in experimentally infected chickens. Vet Microbiol 1995;45:219-31.
14. Belthoff JR, Gauthreaux SA. Partial migration and differential winter distribution of house finches in the eastern United States. Condor 1991;93:374-82.
